# Improving the sleep quality of people regularly taking opioid agonists: a systematic review and meta-analysis of interventions for non-respiratory sleep outcomes

**DOI:** 10.1192/j.eurpsy.2026.11988

**Published:** 2026-07-31

**Authors:** A. Prabhu, J. D. King, C. Moderie, D. Kurtin, A. Lingford-Hughes, L. Paterson

**Affiliations:** 1 Institute of Psychiatry Psychology and Neuroscience, King’s College London; 2 Division of Psychiatry, Imperial College London, London, United Kingdom; 3 Department of Psychiatry, McGill University, Montreal, Canada; 4 Division of Psychiatry, Imperial College London, London, United Kingdom

## Abstract

**Introduction:**

Poor sleep quality is commonly reported by people regularly taking opioid agonists, usually in the context of chronic pain management or as substitution therapy for opioid-use disorder. This may be explained by pharmacological effects of the opioid itself (including respiratory depression and changes in sleep architecture) or by confounding factors including chronic pain, polysubstance misuse, personality traits and co-occurring mental and physical ill-health. Poor sleep quality predicts a poor quality of life, worse mental health, higher average opioid dose, relapse into recreational opioid use, and is a barrier to opioid discontinuation. Improving sleep quality for people regularly taking opioid agonists is therefore a clinical priority.

**Objectives:**

This systematic review aims to synthesise the evidence on the efficacy of interventions to improve sleep in people regularly taking opioid agonists.

**Methods:**

This review was prospectively registered (PROSPERO: CRD42023464386). Structured searches for interventional studies among adults on a stable dose of an opioid agonist were conducted up until July 2025 in multiple databases. Single case reports were excluded, as were people with solely recreational use. Only studies using validated sleep measures were included. Study quality was assessed with standardised risk of bias tools, and unreported data was sought systematically from authors. Meta-analysis was conducted where 3 or more studies examined comparable interventions on comparable populations.

**Results:**

A variety of interventions ranging from treatments recommended for insomnia in the general population such as Cognitive Behavioural Therapy for Insomnia and sedating antidepressants, to herbal treatments and Chinese traditional medicine procedures were included. There were a great number of incomparable trialled interventions, albeit some of which are well conducted large randomised trials, with a small evidence base for each. Meta-analysis suggests trazodone may be a promising intervention. Overall, the evidence of specific treatments for sleep disturbance in opioid users is weak. There may be a more prominent role for psychological and social interventions such as CBT-I and peer activity groups to avoid adverse pharmacotherapy effects.

**Conclusions:**

Given the high prevalence and impact of sleep disturbance in opioid users, future high-quality studies in this area are needed. There is only preliminary evidence to support the use of a range of pharmacotherapies, switching to buprenorphine based substitution therapies, psychosocial interventions or traditional medicine procedures to improve sleep disturbance in regular opioid users.

**Disclosure of Interest:**

None Declared

